# Using built environment characteristics to predict walking for exercise

**DOI:** 10.1186/1476-072X-7-10

**Published:** 2008-02-29

**Authors:** Gina S Lovasi, Anne V Moudon, Amber L Pearson, Philip M Hurvitz, Eric B Larson, David S Siscovick, Ethan M Berke, Thomas Lumley, Bruce M Psaty

**Affiliations:** 1 Institute for Social and Economic Research and Policy, Columbia University, New York, NY, USA; 2 Urban Design and Planning, University of Washington, Seattle, WA, USA; 3 Architecture, Landscape Architecture, University of Washington, Seattle, WA, USA; 4 Department of Geography, University of Washington, Seattle, WA, USA; 5 Center for Health Studies, Group Health Cooperative, Seattle, WA, USA; 6 Department of Epidemiology, University of Washington, Seattle, WA, USA; 7 Department of Medicine, University of Washington, Seattle, WA, USA; 8 Community and Family Medicine, Dartmouth Medical School, Hanover, NH, USA; 9 Department of Biostatistics, University of Washington, Seattle, WA, USA; 10 Department of Health Services, University of Washington, Seattle, WA, USA

## Abstract

**Background:**

Environments conducive to walking may help people avoid sedentary lifestyles and associated diseases. Recent studies developed walkability models combining several built environment characteristics to optimally predict walking. Developing and testing such models with the same data could lead to overestimating one's ability to predict walking in an independent sample of the population. More accurate estimates of model fit can be obtained by splitting a single study population into training and validation sets (holdout approach) or through developing and evaluating models in different populations. We used these two approaches to test whether built environment characteristics near the home predict walking for exercise. Study participants lived in western Washington State and were adult members of a health maintenance organization. The physical activity data used in this study were collected by telephone interview and were selected for their relevance to cardiovascular disease. In order to limit confounding by prior health conditions, the sample was restricted to participants in good self-reported health and without a documented history of cardiovascular disease.

**Results:**

For 1,608 participants meeting the inclusion criteria, the mean age was 64 years, 90 percent were white, 37 percent had a college degree, and 62 percent of participants reported that they walked for exercise. Single built environment characteristics, such as residential density or connectivity, did not significantly predict walking for exercise. Regression models using multiple built environment characteristics to predict walking were not successful at predicting walking for exercise in an independent population sample. In the validation set, none of the logistic models had a C-statistic confidence interval excluding the null value of 0.5, and none of the linear models explained more than one percent of the variance in time spent walking for exercise. We did not detect significant differences in walking for exercise among census areas or postal codes, which were used as proxies for neighborhoods.

**Conclusion:**

None of the built environment characteristics significantly predicted walking for exercise, nor did combinations of these characteristics predict walking for exercise when tested using a holdout approach. These results reflect a lack of neighborhood-level variation in walking for exercise for the population studied.

## Background

Environments that make walking feasible and appealing have been labeled as "pedestrian-oriented" [1] or "walkable" [2]. Such environments may help local residents to maintain active lifestyles and to avoid health conditions for which sedentary behavior is a known risk factor, including obesity, diabetes, cardiovascular disease, and some types of cancer [3]. Residential density, connectivity, land use mix, facilities, paths, and aesthetic features have all been studied as predictors of walking or physical activity [1,2,4-12], but results for these studies have not been consistent. Residential density and connectivity, for example, are associated with walking or physical activity in some studies [13-15], but not in others [16,17]. Unexpected but significant findings have been reported as well, including more walking or physical activity in neighborhoods with reduced access to shops [18,19], fewer physical activity facilities [20,21], or poor sidewalk conditions [22].

Discordance among studies may be due to differences in populations, disagreement between perceptions and objective measures of the environment, or environmental measurement at aggregate levels that mask relevant small-scale variation [1,4,9]. More specifically, individuals may respond differently to their environment depending on their age, affluence, car ownership, physical functioning, attitudes, preferences, or other traits. The differences between perceived neighborhood characteristics and objectively measured characteristics are potentially problematic because being active may change one's perceptions of the environment, making it difficult to separately identify the effect of environmental characteristics on activity. On the other hand, perceptions and objective characteristics may differ because the area measured through "objective" geographic data do not reflect the environment as experienced [23,24], either because the scale is too large or because the shape is not customized to reflect pertinent social or physical boundaries; objective is placed in quotes here because this term has been used to indicate that the data have come from an external source such as a government agency or commercial firm, sources which may themselves provide imprecise or biased data.

Associations between built environment characteristics and physical activity may also depend on the precision or nature of the physical activity measurement. It is important to note that the built environment characteristics that have been used to assess neighborhood walkability may influence walking as a mode of transportation [2,7,25,26]; our study, on the other hand, evaluates whether these characteristics are associated with walking for exercise. Characteristics of the built environment are most strongly correlated with transportation-related activities, especially walking and biking, that occur within the environmental context of study [17,19,27-29]. However, measures of leisure-time physical activity, including walking for exercise or recreational purposes, may also warrant attention because of the link between leisure-time physical activity and health. In our study, walking for exercise was measured because of the established association between regular or brisk walking and cardiovascular health [30-33]. Prior studies of walking for exercise or other leisure-time physical activity have provided some support for the relevance of residential density, street connectivity, sidewalk availability, proximity to potential destinations or fitness centers and parks for these outcomes [1,22,34-40]. However, one should note that the built environment characteristics we measured may have public health relevance through a pathway that does not include walking for exercise.

A final source of inconsistency among published associations of built environment characteristics with physical activity warrants attention: multiple testing or empirically driven model building that could inflate type I errors (false positive associations). Even in the setting of careful measurement, the potential for false positive findings is increased by the practice of screening numerous built environment characteristics for positive associations, and publishing these without independent replication. One way studies of the built environment and health might limit false positive associations is through the creation and validation of a walkability model or index combining several built environment characteristics to optimally predict walking [27,41-43]. The common practice of developing and testing models using the same data, however, could lead to overestimating model fit and prediction [44]. More accurate estimates of model fit can be obtained by splitting a single study population into training and validation sets (holdout approach) or through developing and testing models in different populations [45]. A holdout approach involves exploration and model fitting for a random selection of the study data, called the training set. The remaining data, called the validation or test set, is reserved for replication of the initial results, and to estimate of how well the model would fit an independent sample of data. False positive findings are unlikely to be replicated in the validation set.

In this study, data for a healthy population in Washington State were analyzed using a holdout approach. Our objective was to evaluate whether built environment characteristics near the home could be used to predict walking for exercise. We created models using built environment characteristics near each participant's home address to predict walking for exercise, and then evaluated these models on a random subset of the study data. We also evaluated models based on data from a previously described study using a different sampling frame within the same region [27,42].

## Materials and methods

### Study setting and population

Data came from the Heart and Vascular Health (HVH) study, an ongoing population-based case-control study in the Puget Sound Region of Washington State [46]. Subjects lived in King, Kitsap, Pierce, Snohomish, and Thurston counties; King County, the most populous of these, contains the City of Seattle. Although much of the land area included is rural, 97 percent our study population lived in non-rural areas (defined as a residential density ≥ 96.5 units/km^2 ^[250 units/mi^2^] [47]).

The HVH study was designed for investigating pharmacological and genetic influences on cardiovascular disease, but we used data on 1,608 control participants to examine the effects of the built environment on walking for exercise. The controls from this study were a stratified random sample of 30 to 79 year old members of Group Health, a large health maintenance organization serving approximately 500,000 Washington State residents. Participants gave informed consent, and the human subjects review committee at Group Health and the University of Washington approved all study procedures.

Only controls were included in this analysis, to limit possible recall bias or confounding by preclinical cardiovascular disease. Participants were also excluded if they had a documented history of myocardial infarction, stroke, congestive heart failure or angina, or if they reported fair or poor health prior to their reference date. These exclusions were designed to identify a healthy population in which physical activity might be important for primary prevention of disease, while excluding those with major health limitations that could influence both place of residence and physical activity patterns.

We randomly assigned each participant a reference date within the year of selection as a control (1995 to 2001). Information preceding the reference date was collected from medical records and telephone interviews; the reference date was used by the original study to ensure comparable data quality for myocardial infarction cases and frequency matched controls. Telephone interviews took place from 1995 to 2004, an average of about two years (standard deviation: 0.7 years) after the assigned reference date; 76 percent of eligible, contacted controls agreed to participate in a telephone interview. Compared with participants who allowed us only to examine their medical record, participants completing the telephone interview were more likely to have treated hypertension, treated diabetes, or a body mass index above 30 and less likely to be residents of King County (chi-squared test *p *< 0.05).

### Physical activity and participant characteristics

The telephone interview included questions on physical activity derived from the Minnesota Leisure-Time Physical Activity questionnaire [48]. The Minnesota Leisure-Time Physical Activity questionnaire has a high test-retest reliability [49] for physical activity over the last year, with one month interval between tests, but has been modified for our study. Participants in the HVH study were asked to report the frequency and duration of their participation in 26 types of physical activity, including "walking for exercise", for a one-month period before their reference date. Frequency and average duration were used to estimate the minutes per week spent walking for exercise. Previous studies have found that data from this questionnaire on physical activity or walking for exercise are associated with incident myocardial infarction in this study population [46,50], which suggests the modified questionnaire has predictive validity and relevance to cardiovascular health.

The telephone interview also included questions on the participant's race, general health status (classified as excellent, very good, good, fair, or poor), smoking status, employment status, education, and income. Data from Group Health medical and pharmaceutical records were used to assess whether each participant had treated hypertension or treated diabetes. Measured height and weight were taken from the medical record and used to calculate body mass index (weight in kilograms/height in meters, squared). Obesity was defined as a body mass index above 30.

### Addresses and geocoding

Residential addresses were obtained from Group Health's archived end-of-year membership files for the December before each participant's reference date. An automated process in Maptitude software [51], version 4.7 (Caliper Corporation, Newton/MA, 2004), successfully geocoded 97 percent of addresses, and an additional two percent were geocoded following manual cleaning of the address data. Participants were excluded if they had no address or only a Post Office box available (n = 79); an address that could not be geocoded (n = 4); or an address located outside of the five-county study area (n = 72).

One-kilometer airline buffers (circles with one kilometer radius surrounding each address) were created using ArcView 3.2 (ESRI, Redlands/CA, 1999). Airline buffers based on Euclidean distance were used instead of network buffers based on empirical evidence from the same geographic region [52] and the high permeability of urban environments to pedestrians [53]. One kilometer buffers were selected because of the relatively small territory typically covered on foot [8,29] and the lack of correlation between perceived and objective measures of the built environment beyond one kilometer [20,42].

Addresses were also allocated to census block groups, census tracts and ZIP codes using a point-in-polygon joining process [54]. Census block groups in the US contain approximately 1,000 residents, census tracts 4,000 residents, and ZIP codes 30,000 residents [55].

### Built environment data

For each of the five study counties digital maps of street networks, parks, and tax parcels (defined as buildings or units of land that are taxed or exempt from taxation) were obtained through the Washington State Geospatial Data Archive [56], county agencies, or cities (sidewalks, for King County only). Built environment data sources used were produced between 1998 (the midpoint of the study period) and 2005; although data from 1998 were sought in all cases, more recent data were used for several built environment characteristics because older data had not been archived, were of poor quality, or did not exist for a given county.

Residential density was calculated as housing units per square kilometer, with a housing unit defined as a house, apartment, mobile home, or other dwelling intended for occupancy as separate living quarters [57]. Residential density of each one-kilometer buffer was estimated using an area-weighted average of densities from census block groups intersecting or contained in the buffer. For example, a subject might have 30 percent of their one-kilometer buffer in census block group A, and 70 percent in census block group B. The estimated density for the one-kilometer buffer would then be 0.3 * (density of A) + 0.7 * (density of B). As a measure of connectivity, block size was calculated using local street maps. For sidewalk availability, the total length of sidewalk-lined streets within each one-kilometer buffer was calculated. Sidewalk data were only available for King County.

We estimated proximity to several potential walking destinations (grocery stores, schools, restaurants and bars, banks, grocery-restaurant-retail complexes, office complexes, school-church combinations, fitness facilities, and parks), calculating the distance to the closest destination of each type and the number of destination of each type within one kilometer. For the destination combinations (grocery-restaurant-retail complexes, office complexes, and church-school combinations), the area of the nearest one was also calculated. Park access was measured as the proportion of the one-kilometer buffer covered by parks. With the exception of parks, which were identified using digital maps of parks in each county, destinations were identified using tax parcel land use codes. The categorization of the land use codes differed by county, but consistent rules were applied to categorize land uses across counties.

### Statistical analysis

Built environment characteristics were tested as predictors of walking for exercise. All participants were included in analyses of logistic models predicting some walking versus no walking, and those who walked were included in linear models to predict amount of walking (average minutes per week). Time spent walking for exercise was log-transformed to moderate the effects of skewness and heteroscedasticity.

We tested single built environment characteristics and models using multiple built environment characteristics to predict walking. Some built environment characteristics may be associated with walking in our sample by chance alone, raising concerns about multiple comparisons. If we fit a model to our data, and then tested the model using the same data, our estimates of model fit would be artificially high because any chance associations unique to our data would be incorporated into our model. This would overestimate our ability to predict walking in a different sample of individuals from the same population. A holdout approach was used to avoid this bias [44,45]. Models developed in a training set were tested in a validation set, with estimates of model fit based on the validation set considered to be more accurate.

The training set (a stratified random sample of 2/3 of participants) and validation set (the remaining 1/3 of participants) were similar with regard to demographic, socioeconomic, health, and built environment characteristics. The random sampling was stratified by King County residence, because we decided *a priori *to separately create and evaluate models for the subset that lived in King County, in addition to pooled models for the entire region. More than half of area residents and a majority of our study participants (58%) lived in King County.

Built environment characteristics were modeled within categories or log-transformed in order to reduce the influence of outliers. Proximity to destinations of each type was categorized as within 500 m, 500 m to 1000 m, or more than 1000 m away. Density, connectivity, sidewalk availability, and park access were log transformed. Regression models were used to calculate the predicted probability of walking for exercise or predicted minutes per week of walking for exercise. These predicted variables were proportional to the linear predictors from the corresponding models: a constant (alpha) added to the product of each built environment characteristic (x) and the corresponding slope parameter (beta coefficient): predicted minutes/week of walking = α + Σx_i_β_i_. Slope parameters were estimated from training set data.

In addition, models were created using the Walkable and Bikeable Community (WBC) study model components: residential density; household and average block size; sidewalk availability; number of schools, restaurants or bars, grocery stores, and grocery-restaurant-retail complexes; distance to the closest restaurant or bar; distance to the closest grocery store; and area of the closest office complex [27,42]. We evaluated regression models with slope parameters for these 11 characteristics based our study's training set or on the WBC study data [27,42] (reanalyzed with exclusions, adjustments, and regression techniques parallel to those used for the present study).

For logistic regression models, model fit was evaluated using Hosmer-Lemeshow tests [45] and C-statistics (based on the area under the receiver operating characteristic curve). Under the null hypothesis, the logistic model predicts walking no better than expected by chance, and one would expect a C-statistic of 0.5; a model with perfect prediction would lead to a C-statistic of 1.0. Predictive utility of linear models was assessed through the percent of variation explained: r-squared * 100 percent.

Unadjusted models were compared with models adjusted for age, sex, self-reported health status, income, and education. For adjusted models, missing values for income (10 percent) and education (less than one percent) were estimated through multiple imputation [58]. Because unadjusted and adjusted models were similar, we have presented the unadjusted models in our tables. All regression models were run using robust variance estimates in Stata 8.2 (StataCorp, College Station/TX, 2003), and variance estimates accounted for clustering within county of residence.

Intra-class correlation coefficients (ICCs) were used to evaluate how characteristics varied between versus within ZIP codes, census tracts, and census block groups [59]. These ICCs can be interpreted as the maximum proportion of variation explained at the given group-level. If a characteristic was constant within each group, the only variation would be between groups and the ICC would be 1.0. In contrast, if the characteristic was randomly distributed with respect to group, the ICC would be close to zero. These estimates were based on one-way analysis of variance (ANOVA) models. Continuous variables were log-transformed to more closely meet the normality assumption of the ANOVA model. The ANOVA ICC estimator was also used for dichotomous variables, for which the ICC estimation remains asymptotically valid and unbiased [60].

## Results

For 1,608 participants meeting the inclusion criteria, the mean age was 64 years, 61 percent were female, 90 percent were white, 37 percent had a college degree, and 46 percent were retired. The annual household income was above $50,000 for 51 percent of non-retired participants and 21 percent of retired participants.

Sixty-two percent of participants reported that they walked for exercise (Table 1). Older participants and women were more likely to report walking for exercise. Even after excluding participants in fair or poor health, general self-reported health status was associated with walking. Among those who reported walking for exercise, the median walking time was 2.3 hours per week (interquartile range: 1.4 to 3.6 hours per week) and the mean walking time was 2.9 hours per week (standard deviation: 2.5 hours per week).

**Table 1:** Characteristics of participants who did and did not walk for exercise

	**Did not walk for exercise**	**Walked for exercise**
	N = 608	N = 1,000

Age, %		
30 to 45	3	2
45 to 55	21	18
55 to 65	28	27
65 to 75	33	35
75 to 79	15	17
Female sex, %	56	64
White race, %	89	91
Self-reported health status, %		
Excellent	17	19
Very good	34	39
Good	49	41
Treated hypertension, %	72	67
Treated diabetes, %	9	8
Obese (body mass index > 30), %	42	35
Current smoking, %	14	7
Retired, %	41	49
Income, %		
< $25,000/yr	22	22
$25,000 to $50,000/yr	40	40
> $50,000/yr	38	38
Education, %		
High school or less	31	28
Some college/college graduate	51	54
Graduate/professional	18	18
County of residence, %		
King	57	59
Kitsap	7	6
Pierce	11	9
Snohomish	15	15
Thurston	9	12

We evaluated single built environment characteristics, including residential density, street connectivity, sidewalk availability, proximity to destinations, and park access, as predictors of walking for exercise. Density of housing units had a C-statistic of 0.52 (95 percent confidence interval: 0.49, 0.55) for predicting walking versus no walking and explained less than 0.1 percent of the variation in walking time (Table 2). Connectivity, measured by block size, had a C-statistic of 0.49 (95 percent confidence interval: 0.46, 0.51) and explained 0.6 percent of walking time. Sidewalk availability, measured only in King County, had a C-statistic of 0.51 (95 percent confidence interval: 0.47, 0.54) and explained 0.1 percent of the variation in walking time. Similarly modest results were found for other single measures, such as proximity of the various destinations (Table 2). A more general measure of proximity to potential destinations (proportion of the one-kilometer buffer occupied by commercial buildings) was also considered, but was not significantly associated with walking for exercise.

**Table 2:** Built environment characteristics used one at a time to predict walking for exercise

**Built Environment Characteristic**	**Median** (Interquartile range)	**Walking or not** C-statistic (95% CI)	**Walking time** Proportion of variation explained
Density (thousands of residential units per km^2^)	177 (105, 256)	0.52 (0.49, 0.55)	0.0 %
Connectivity (mean block size in km^2^)	0.13 (0.04, 0.44)	0.49 (0.46, 0.51)	0.6 %
Sidewalks^a ^(km of sidewalk-lined streets)	12 (1, 33)	0.51 (0.47, 0.54)	0.1 %
Destinations (count within 1 km buffer)			
Grocery stores	1 (0, 5)	0.50 (0.46, 0.52)	0.0 %
Restaurants	3 (0, 11)	0.50 (0.48, 0.53)	0.1 %
Retail stores	8 (0, 26)	0.51 (0.48, 0.54)	0.0 %
Grocery-restaurant-retail complexes	0 (0, 1)	0.52 (0.50, 0.54)	0.0 %
Offices	6 (1, 23)	0.52 (0.49, 0.54)	0.2 %
Office complexes	0 (0, 1)	0.52 (0.49, 0.54)	0.0 %
Banks	1 (0, 7)	0.49 (0.46, 0.52)	0.1 %
Churches	4 (1, 12)	0.48 (0.44, 0.51)	0.1 %
Schools	4 (1, 9)	0.50 (0.47, 0.53)	0.2 %
School-church combinations	0 (0, 1)	0.50 (0.48, 0.53)	0.2 %
Fitness centers	1 (0, 5)	0.49 (0.46, 0.52)	0.1 %
Parks (percent of 1-km buffer covered)	2 (0, 5)	0.51 (0.49, 0.54)	0.5 %

Notes: Built environment characteristics were measured within a one-kilometer airline buffer; ^a ^Sidewalk data were available only for King County, and were investigated as predictors of walking in this subset

Built environment characteristics were then combined to create linear and logistic models predicting walking for exercise, to be validated using a holdout approach. Parameter estimates from logistic and linear models fitted to the training set are shown in Table 3 (un-italicized estimates). Several built environment characteristics were significantly associated with walking for exercise in the training set (indicated by bold text). The training set models using this bloc of predictors were evaluated using a holdout approach, with the corresponding measures of model fit shown at the top of Table 4.

**Table 3:** Models using multiple built environment characteristics to predict walking for exercise

**Built Environment Characteristic**	**Logistic model of walking or not**		**Linear model of walking time**	
	Training set β estimate	*Validation set β estimate*	Training set β estimate	*Validation set β estimate*
**Density**				
log(thousands of residential units/km^2^)	0.25	*-0.33*	**0.07**	*-0.13*
**Connectivity**				
log(mean block size in km^2^)	0.01	*-0.04*	0.04	*0.03*
**Sidewalks**				
log(kilometers of sidewalk-lined streets)	**-0.08**	***0.16***	**0.06**	*-0.03*
**Destinations**				
Distance to closest bank				
< 500 m	1.00 (ref)	*1.00 (ref)*	1.00 (ref)	*1.00 (ref)*
500 to 1000 m	0.09	***-0.49***	-0.11	*-0.01*
> 1000 m	0.00	*0.25*	-0.12	*0.13*
Distance to closest church				
< 500 m	1.00 (ref)	*1.00 (ref)*	1.00 (ref)	*1.00 (ref)*
500 to 1000 m	-0.29	***-0.56***	0.03	*0.17*
> 1000 m	-0.21	*-0.49*	0.04	*0.06*
Distance to closest school				
< 500 m	1.00 (ref)	*1.00 (ref)*	1.00 (ref)	*1.00 (ref)*
500 to 1000 m	-0.20	*0.21*	**-0.22**	*-0.23*
> 1000 m	-0.16	*0.27*	**-0.26**	*-0.22*
Distance to closest grocery store				
< 500 m	1.00 (ref)	*1.00 (ref)*	1.00 (ref)	*1.00 (ref)*
500 to 1000 m	-0.05	*0.11*	0.14	***0.37***
> 1000 m	**-0.39**	*-0.07*	0.06	***0.20***
Distance to closest office				
< 500 m	1.00 (ref)	*1.00 (ref)*	1.00 (ref)	*1.00 (ref)*
500 to 1000 m	0.17	***0.90***	-0.02	*0.18*
> 1000 m	**0.34**	*0.28*	**0.20**	*0.06*
Distance to closest retail shop				
< 500 m	1.00 (ref)	*1.00 (ref)*	1.00 (ref)	*1.00 (ref)*
500 to 1000 m	**-0.44**	*0.26*	0.01	*-0.46*
> 1000 m	-0.25	*-0.13*	-0.11	*-0.04*
Distance to closest restaurant				
< 500 m	1.00 (ref)	*1.00 (ref)*	1.00 (ref)	*1.00 (ref)*
500 to 1000 m	**0.42**	*-0.13*	0.05	*0.08*
> 1000 m	-0.01	*0.26*	0.07	*-0.18*
Distance to closest gro-rest-ret complex				
< 500 m	1.00 (ref)	*1.00 (ref)*	1.00 (ref)	*1.00 (ref)*
500 to 1000 m	-0.18	*0.07*	**-0.13**	*-0.08*
> 1000 m	**-0.34**	*-0.08*	-0.03	*-0.15*
Distance to closest office complex				
< 500 m	1.00 (ref)	*1.00 (ref)*	1.00 (ref)	*1.00 (ref)*
500 to 1000 m	**0.14**	***-0.69***	0.03	*0.01*
> 1000 m	0.25	*-0.49*	0.01	*-0.11*
Distance to closest school-church combo				
< 500 m	1.00 (ref)	*1.00 (ref)*	1.00 (ref)	*1.00 (ref)*
500 to 1000 m	0.03	*0.23*	-0.01	*0.16*
> 1000 m	0.03	*0.04*	0.08	*0.00*
Distance to closest fitness center				
< 500 m	1.00 (ref)	*1.00 (ref)*	1.00 (ref)	*1.00 (ref)*
500 to 1000 m	-0.01	*-0.10*	-0.08	*0.12*
> 1000 m	0.03	*0.10*	-0.02	*0.08*
**Park area**				
log(proportion of area covered by park)	-0.04	*0.12*	**0.06**	***0.11***

Notes: The outcomes were walking for exercise versus not walking for exercise and log(minutes per week walking for exercise); *italics *have been used for the validation set estimates to emphasize that these were post hoc and for comparison only; **bold **text has been used to indicate statistical significance (p < 0.05)

**Table 4:** Holdout validation and replication of models using the built environment to predict walking for exercise

	Logistic model of walking or not		Linear model of walking time	
	Training set, C-statistic (95% CI)	Validation set, C-statistic (95% CI)	Training set, variation explained	Validation set, variation explained
Models from Table 3, training set β estimates	0.58 (0.55, 0.62)	0.46 (0.41, 0.51)	4.1 %	0.0 %
Restricted to King County	0.61 (0.57, 0.66)	0.45 (0.38, 0.52)	5.6 %	0.2 %
WBC components, training set β estimates	0.56 (0.53, 0.60	0.46 (0.41, 0.51)	1.9 %	0.3 %
Restricted to King County	0.58 (0.53, 0.62)	0.44 (0.38, 0.51)	3.2 %	0.3 %
	Training and validation sets, C-statistic (95% CI)		Training and validation sets, variation explained	
WBC components, WBC β estimates	0.50 (0.48, 0.53)		0.1 %	
King County only	0.51 (0.47, 0.55)		0.1 %	

Notes: All models were run for the entire HVH population and restricted to King County residents; C-statistic indicates area under the receiver operating characteristic curve; CI indicates confidence interval; WBC indicates Walkable and Bikeable Communities Study

In the training set, the logistic regression model shown in Table 3 had an overall C-statistic of 0.61 (95 percent confidence interval: 0.58, 0.65) for predicting some walking for exercise versus none (Table 4, top). A Hosmer-Lemeshow test showed that the expected and observed numbers of walkers were similar across deciles of predicted probability of walking, so that the logistic regression model was not significantly rejected for the training set on the basis of this goodness-of-fit test. In the training set, the linear regression model predicted about four percent of the variation in walking time (Table 4, top).

In accordance with the planned holdout approach, models with parameter estimates based on the training set were evaluated in the validation set to more accurately estimate how well they would predict walking in a new sample of individuals from the same population. When the logistic model with parameters based on the training set was used to predict walking in the validation set, the C-statistic estimate had a confidence interval that included the null value of 0.5 and the percent of variation explained by the linear model was less than one percent (Table 4, top). In the validation set, the Hosmer-Lemeshow test indicated that the model did not fit the data well: across deciles of predicted walking probability, the expected and observed numbers of walkers were significantly different (*p *< 0.001). The pattern observed in the validation set data significantly deviated from what was expected based on the model fitted to the training set data.

In a post hoc analysis, we created logistic and linear models with parameters based on the validation set (Table 3, italicized estimates). While some parameters were similar for the training and validation sets, others were significant in each model but of opposite sign. For example, sidewalk availability was associated with a lower probability of walking in the training set but a higher probability of walking in the validation set.

Logistic and linear models using the same bloc of built environment characteristics were also estimated for the King County residents only or with adjustment for potential confounders. When restricted to King County residents, estimates of model fit in the training set were even higher (Table 4).

The models tested using the holdout approach may have failed in the validation set because they incorporated so many variables; the number of variables increases the probability that the model will overfit the training set data, explaining random noise unique to the data. In order to address this concern, we repeated the process of model fitting and model evaluation with a smaller number of variables, selected based on their inclusion in the models from the WBC study [27,42]. When considering the 11 components of the WBC study models (Table 5), the direction of association was not consistent for these built environment characteristics between the models in the HVH study population and those from the WBC study. Neither models with training set parameter estimates nor those with WBC parameter estimates significantly predicted the corresponding walking outcome outside of the sample in which it was fitted (Table 4).

**Table 5:** Built environment characteristics associated with walking (WBC study) or walking for exercise (HVH study)

**Built Environment Characteristic**	**Logistic model of walking or not**		**Linear model of walking time**	
	WBC β estimate	HVH β estimates	WBC β estimate	HVH β estimate

**Density**				
log(dwellings/acre within buffer)	0.16	0.18	**0.25**	0.01
**Connectivity**				
grouped linear household block size	-0.07	-0.02	-0.02	0.00
log(average block size in buffer)	-0.09	0.05	0.08	0.04
**Sidewalks**				
miles of sidewalk in buffer	-0.04	**-0.01**	0.02	0.00
**Destinations**				
grouped linear num grocery stores	-0.27	0.03	-0.06	-0.01
log(dist to closest grocery store)	**-0.68**	**0.19**	0.01	**-0.13**
number of bars/restaurants	0.02	0.00	0.01	-0.00
log(dist to closest bar/restaurant)	-0.16	0.01	-0.03	-0.05
num gro-rest-retail complexes	**0.27**	**0.14**	0.06	-0.00
log(area of closest office complex)	-0.09	-0.01	**-0.05**	**0.03**
log(number of educational parcels)	-0.23	-0.08	-0.09	**-0.11**

Notes: WBC indicates Walkable and Bikeable Communities Study; HVH indicates Heart and Vascular Health Study

Selecting variable transformations that maximized the model fit in the training set, adjusting for potential confounders (sex, age, health status, education and income), or restricting to non-rural areas did not improve model fit in the validation set. Models using the same built environment characteristics also failed to reliably predict total physical activity time per week [61], a measure described elsewhere [46,50].

To better understand these results, the geographic variation in walking for exercise and three continuous measures of the built environment (density, connectivity, and park area) were explored using ICCs (Table 6). Small correlations were observed within census areas for amount of walking for exercise but these were not significant. Since the ICC confidence intervals for both walking measures included 0.000, the data were compatible with no neighborhood-level pattern in walking for exercise. Residential density, connectivity, and park area were highly correlated within census tracts and census block groups, as expected.

**Table 6:** Geographic variation in physical activity and the built environment

	**ZIP code**	**Census tract**	**Census block group**
	ICC (95% CI)	ICC (95% CI)	ICC (95% CI)
**Walking for exercise**			

Some versus none	0.003 (0.000, 0.025)	0.000 (0.000, 0.051)	0.000 (0.000, 0.102)
Minutes per week^ab^	0.000 (0.000, 0.036)	0.046 (0.000, 0.129)	0.055 (0.000, 0.211)

**Built environment characteristics**			

Residential density of 1-km buffer^a^	0.739 (0.680, 0.799)	0.920 (0.907, 0.932)	0.953 (0.946, 0.960)
Connectivity of 1-km buffer^a^	0.784 (0.731, 0.836)	0.879 (0.861, 0.897)	0.931 (0.920, 0.941)
Park area within 1-km buffer^a^	0.330 (0.255, 0.405)	0.601 (0.554, 0.648)	0.778 (0.746, 0.809)

Notes: ICC indicates intra-class correlation coefficient; CI indicates confidence interval; except where otherwise indicated N = 1,608

^a ^Log-transformed to approximate normality

^b ^Among participants reporting some walking for exercise: N = 1,000

## Discussion

In this study, built environment characteristics were measured within one kilometer of participants' residential addresses, but these built environment characteristics were not consistent predictors of walking for exercise. Models using these built environment characteristics to predict walking for exercise could not be validated using a holdout approach. This was true for the outcomes of walking for exercise versus not walking for exercise or amount of walking for exercise among those who walked; for models with parameters estimated from a random sample of the study data or parameters estimated from a different study population in the same geographic region; for analyses restricted to the most populous county or to non-rural areas; and for models with and without adjustment for potential confounders. Participants living in the same census block group, census tract, or ZIP code were no more similar with respect to walking for exercise than would be expected by chance. This lack of significant neighborhood-level variation in physical activity variables was found despite the presence of neighborhood-level variation in residential density, connectivity, and park area.

This study suggests that the amount of walking for exercise explained by the objectively measured built environment characteristics near one's home may be quite small, possibly accounting for one percent of the total variation. The importance of immediate physical surroundings may be limited because of the many social and psychological influences shaping physical activity behavior [39,62,63]. The larger estimates of model fit from the training set did not reflect how well the models would predict walking for exercise in an independent sample of the same population.

The physical activity data used in this study were collected for their relevance to cardiovascular disease [46,50]. Walking for transportation or walking within one's neighborhood may be more sensitive to the local built environment, but should continue to be evaluated with respect to health outcomes [64,65]; walking pace and validity of self-report may be lower for transportation walking compared with walking for exercise [66] so that the association between transportation walking and improved health outcomes should be tested and not assumed. The present findings do not directly address the hypothesis that built environment characteristics influence walking for transportation, which has been separately evaluated in the same geographic region [67] and elsewhere [7,14,25,68-70].

Previous studies that measured walking for different purposes found different neighborhood determinants of walking for transportation versus recreational purposes [2,9,22,27,28,35,71,72], and the neighborhood built environment has been more strongly associated with walking for transportation as compared with walking for exercise or recreation. While expert consensus [39] and some previous studies [20,34,37,38,71-73] support an association between the neighborhood built environment and walking for recreation or exercise, findings from the present study agree with studies in other regions and populations that have reported no association between the built environment and walking for exercise or recreation [16,21,22,28].

More than half of our study population was age 65 or older. Older adults may be particularly sensitive to their built environment [74,75] and several studies that have focused on the importance of the built environment for supporting the physical activity and independence of older adults. Urban design, the availability of services, recreational facilities, and safety from crime have been associated with more walking in previous studies of older adults [13,34,76,77]. One study of older women found stronger associations between the built environment and pedometer measures, compared with self-reported physical activity, suggesting transport walking may be important [77]. However, older adults may be less likely to walk for transportation [27]. Future research on the importance of walkability for older adults may find pedometer measures to be more sensitive to the built environment. There is also some evidence that walkability may affect the physical activity and health of older adults through increased social capital [78] or social cohesion [79], and understanding the multiple pathways through which the built environment affects health will be important for guiding policy decisions [67].

### Limitations

Data on walking for exercise were derived from telephone interview data, a method subject to recall error [64,80] and social desirability bias [81]. Compared with more vigorous physical activities, walking may be underestimated due to low salience [4,80,82]. Also, the external validity of the present study is limited by the setting and by restrictions chosen to enhance internal validity: all participants had health insurance, participated in a telephone interview, reported good health, had no history of cardiovascular disease, and lived in the Puget Sound Region of Washington State. While these restrictions served to reduce confounding by socioeconomic status or prior health status, they may also have reduced variability. This observational study cannot exclude the possibility that uncontrolled confounding is masking the true relationship between the built environment and walking for exercise.

The measurements of the built environment for this study were based on publicly available data sources. Tax parcel land use codes may have misclassified some relevant destinations, and some built environment characteristics may have changed between the time measurement and the period of physical activity assessment. Some aspects of the local built environment that could influence walking for exercise, such as walking trails, tree cover, landscaping, hills, or building architecture [2,81,83], were not assessed in this study. However, the lack of geographic variation in the outcome, walking for exercise, would limit this study's statistical power to investigate other built environment characteristics. Finally, built environment characteristics were assessed for one-kilometer circular buffers which may not precisely reflect the environment experienced by study participants [23,24].

## Conclusion

Built environment characteristics near home did not consistently predict walking for exercise in this healthy population in western Washington State. Further, there was little evidence of neighborhood-level variation in walking for exercise, despite neighborhood-level variation in the built environment. The built environment may support walking for other specific purposes, such as transportation. The poor prediction of walking for exercise in our study may be due to a weak association between the built environment and walking for exercise, or may reflect the need to incorporate a wider range of built environments by conducting national or international studies [10]. Future research is needed to estimate and confirm the effects of the built environment on different types and measures of walking, physical activity, and health outcomes. Replication across study populations is needed to support accurate predictions, cost-effectiveness analyses, intervention studies, and recommendations for health promotion.

## Abbreviations

Abbreviations used in the text or tables: Heart and Vascular Health (HVH) study, Walkable and Bikeable Community (WBC) study, intra-class correlation coefficient (ICC), analysis of variance (ANOVA), and confidence interval (CI).

## Competing interests

The author(s) declare that they have no competing interests.

## Authors' contributions

All authors contributed to the study design, analytic approach, and presentation of results. The geographic information systems tools and data layers for this project were developed by GSL, AVM, ALP, and PMH. GSL geocoded the study addresses and created neighborhood measures, conducted the analyses, and prepared the manuscript. All authors critically reviewed manuscript drafts and approved the final manuscript.
